# Human Resource Information Systems in Health Care: Protocol for a Systematic Review

**DOI:** 10.2196/resprot.4922

**Published:** 2015-12-01

**Authors:** Aizhan Tursunbayeva, Claudia Pagliari, Raluca Bunduchi, Massimo Franco

**Affiliations:** ^1^ Department of Economics, Management, Society, and Institutions University of Molise Campobasso Italy; ^2^ eHealth Research Group Usher Institute of Population Health Sciences and Informatics University of Edinburgh Edinburgh United Kingdom; ^3^ Business School University of Edinburgh Edinburgh United Kingdom

**Keywords:** eHealth, health care management, information systems, systematic review, human resource information systems

## Abstract

**Background:**

Compared with the eHealth literature as a whole, there has been relatively little published research on the use and impact of information and communication technologies (ICTs) designed to support business functions within health organizations. Human resource information systems (HRISs) have the potential to improve organizational efficiency and effectiveness by facilitating workforce planning, financial and operational administration, staff training, and management analytics. However, the evidence base regarding HRIS in health care is widely distributed across disciplinary boundaries and previous reviews have been somewhat limited in scope. This rigorous systematic review will identify, appraise, and synthesize existing international research on the implementation and impacts of HRIS in health organizations, to provide insights and recommendations that may guide future purchasers, commissioners, implementers, evaluators, and users of such systems.

**Objective:**

The objectives of this review are threefold: (1) to determine the prevalence and scope of existing research and evaluation pertaining to HRIS in health organizations; (2) to analyze, classify, and synthesize existing evidence on the processes and impacts of HRIS development, implementation, and adoption; and (3) to generate recommendations for HRIS research, practice, and policy, with reference to the needs of different stakeholders and communities of practice.

**Methods:**

A high-level scoping review was first undertaken to inform a draft search strategy, which was refined through several cycles of piloting and iteration to optimize its sensitivity and specificity. This was used by the first author, with the help of a medical librarian, to search international electronic databases indexing medical, business, ICT, and multi-disciplinary research. Sources of gray literature and reference lists of included studies were also searched. There were no restrictions on language or publication year. Two reviewers are now screening and coding titles and abstracts for potentially eligible studies, for which full text articles will be retrieved. Reasons for exclusion will be noted for the remaining articles. A structured form will be used to summarize and classify the articles. Any disagreements between reviewers will be resolved through consensus or arbitration by a third reviewer. A PRISMA flow diagram will illustrate the study selection process and ensure transparency of the review. Finally, content experts will be consulted to ensure that important articles have not been missed.

**Results:**

The initial searches have now been completed and the results are being analyzed. The review is expected to be completed and published by the end of 2015.

**Conclusions:**

By synthesizing the existing evidence base, identifying areas in which knowledge is currently lacking, and generating recommendations for research and practice, this review will be a useful resource for decision makers and managers considering or implementing HRIS, as well as encouraging new research in this area.

**Trial Registration:**

PROSPERO International Prospective Register of Systematic Reviews: CRD42015023581; http://www.crd.york.ac.uk/PROSPERO/display_record.asp?ID=CRD42015023581#.VYu1BPlVjDU (Archived by WebCite at http://www.webcitation.org/6ckJCDdCL)

## Introduction

### Background

The health sector is complex [1]; while it must inevitably respond to treatment advances, it can be slow to adopt other types of innovation and has lagged behind other sectors in its use of information and communications technology (ICT) [2]. Health organizations are characterized by a dual structure: one for clinical functions and the other for business and support functions [3], which is reflected in their ICT. Despite the importance of business and support systems in health organizations, very little research on their adoption or impacts exists, compared with other areas in health informatics and eHealth [4]. Like clinical information systems, business and support systems vary depending on the department in which they are used (eg, finance, human resources [HR], or procurement) and may be implemented either as standalone systems (eg, integrated procurement system, or inventory management module) or as a part of generic health information or enterprise resource planning (ERP) systems.

This review focuses on one such functional system, human resource information systems (HRISs), either implemented as a standalone system (eg, a payroll module, or as a dedicated multifaceted HR system), or embedded within a broader generic health information system (eg, as an HR module within an ERP system). Although HRIS are vital for the effective operation of health organizations and address many of the information, communication, and training issues of health professionals [2], they are underrepresented within the health, information systems, and management literature. This is despite the fact that “people costs” can account for 65-80% of health organizations’ total operating budgets [3] and successful implementation of HRIS in HR departments has been linked to improvements in patient care [2].

The importance of HRIS, and the data that they can generate, has also been highlighted by various global health initiatives [5-15]. For example, the World Health Organization (WHO), in their 2006 World Health report stated that “systems for recording and updating health worker numbers often do not exist, which presents a major obstacle to developing evidence-based policies on human resource development” [16]. Six years later, despite the importance of HRIS for underpinning strong health systems, a 2012 review ([17] cited in [18]), concluded that “universal understanding of the HRIS used in monitoring human resources for health is minimal and baseline information regarding their scope and capability is practically non-existent...[there is a need] for more descriptive research of HRIS globally, including the documentation of impact so as to advance the science and evidence-based practice in this area” [18]. Some researchers have already responded to this request and made their contributions to the international peer-reviewed literature [18,19]. However, because this topic lies at the intersection of informatics, management, and health, most of the existing HRIS studies in health care are spread across several discipline-specific bodies of knowledge, making it difficult to obtain a complete picture of the evidence base.

### Formative Scoping

**Figure 1:**
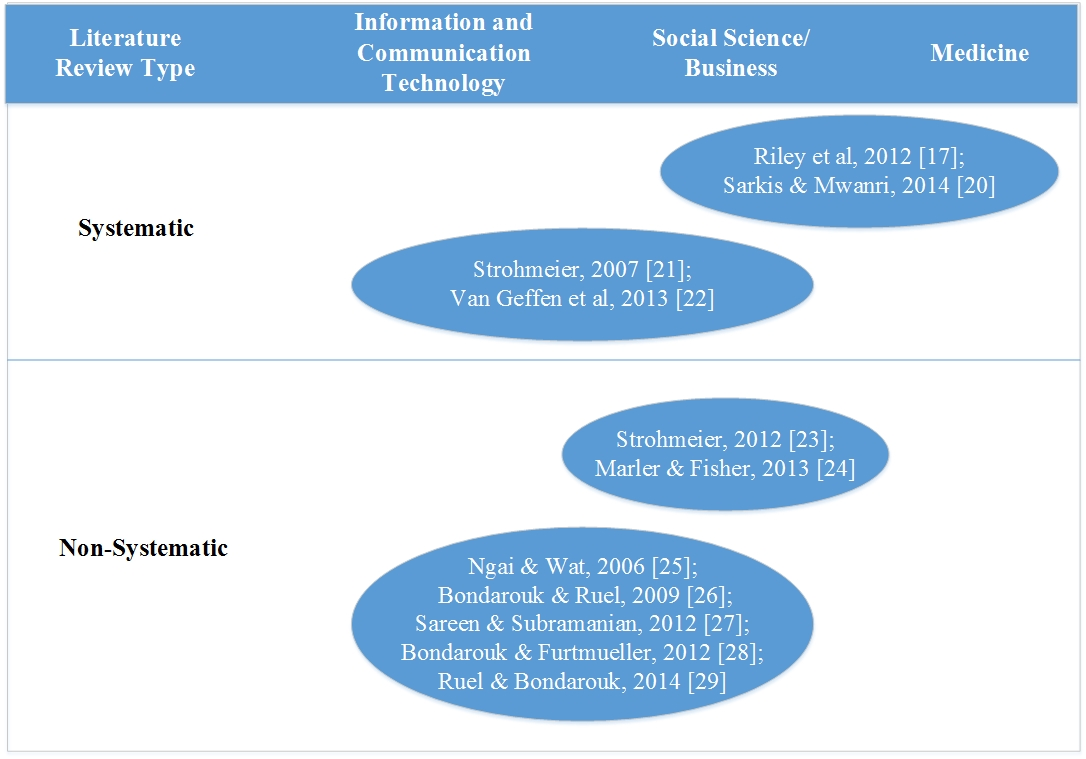
Analysis of existing literature reviews on human resource information systems (HRISs).

To verify the aforementioned claim, we first searched for and examined existing reviews of HRIS literature, and indeed, as can be seen in Figure 1, these tend to be discipline specific, typically coming either from a business, social science, or ICT perspective.

Reviews were classified as systematic if they were either labeled as such, or used a structured search strategy to interrogate named online databases, and clear methods for filtering, summarizing, and synthesizing results. Only a few used a systematic approach [17,20-22], and none encompassed the ICT, social science, business, and health literatures in combination. Only 2 reviews looked specifically at HRIS in health care [17,20], both of which prioritized medical and social science databases, and were limited in scope. The first, published in 2012, was aimed at uncovering baseline information on the use and capability of HRIS in different countries, as a means of understanding the challenges this presents for global health workforce monitoring and development. The second, published in 2013, examined the role of different types of ICTs as a means of enabling continuing professional development to strengthen human resource capacity in health care.

Other nonsystematic literature reviews [23-29] were classified according to their references and the journals in which these references were published. The 6 literature reviews included in Figure 1 [21,22,24,26,28,29] were adopted from “Orchestrating the e-HRM symphony” [30], an inaugural lecture given by Professor T Bondarouk at the University of Twente in December 2014. The other 5 [17, 20, 23, 25, 27] were already known to the authors, based on their background reading.

Our scoping searches, using different keywords, revealed a wide variety of terms and definitions that have been used in the literature to describe information systems aimed at supporting human resource management (HRM) [21], echoing previous scholars’ observations on the lack of consistency and agreement in this area [26]. Such systems may be explicitly referred to as *HRIS* (eg, defined as “the composite of databases, computer applications, and hardware and software necessary to collect/record, store, manage, deliver, present, and manipulate data for human resources” [31]), as an *electronic human resource* or *E-HR* system (eg, described as a “created real-time, information-based, self-service, interactive work environment” [32]) or, more recently, as an *electronic human resource management* or *e-HRM* system (eg, defined as “a way of implementing HR strategies, policies, and practices in organizations through conscious and directed support of and/or with the full use of web-technology-based channels” [33]). These terms are often used to refer to standalone information systems for HR, whereas, in complex organizations, HR functions may be embedded within wider *ERP* systems [34] or *health information systems* [35]. Because the proposed systematic review is designed to be interdisciplinary, comprehensive, and inclusive, it will encompass any study referring to ICT aimed at supporting the administration, management, and development practices of HR in the health sector [36], whether used solely by HR professionals or jointly by HR and health professionals and leaders. This may include such HRM practices as staffing, training and development, compensation and rewards, communication, employee engagement, and performance management [36]. Such systems are not directly related to patient care, although their use in HRM has the potential to indirectly influence care quality and outcomes [37].

### Aims

We are undertaking an interdisciplinary systematic review to identify and classify existing evidence on development, adoption, implementation, and impacts of HRIS on health organizations. In addition to documenting and classifying the research, we will seek to understand the outcomes and implications of HRIS implementation in health organizations, the theoretical frameworks used to study them, and to identify unexplored areas. This review also aims to support health leaders by summarizing the current evidence base and informing recommendations on HRIS implementation and research.

### Objectives

This review concerns the effective development, implementation, and use of ICT platforms and software for supporting the effective management of HR within health organizations and, in some cases, across health care systems. The specific objectives of this review are the following: (1) to determine the prevalence and scope of existing research and evaluation pertaining to HRIS in health organizations; (2) to analyze, classify, and synthesize existing evidence on the processes and impacts of HRIS development, implementation, and adoption; and (3) to generate recommendations for HRIS research, practice, and policy, with reference to the needs of different stakeholders and communities of practice (eg, eHealth, business ICT systems, and HRM).

## Methods

### Design

This is the protocol for a structured review of scientific and gray literature on HRIS in health care. A PRISMA flow diagram [38] will be followed, to illustrate the study selection process and ensure transparency of the review (Trial Registration: CRD42015023581).

### Data Sources and Search Methods

A high-level scoping review was first undertaken to inform a draft search strategy, which was refined through several cycles of piloting and iteration to optimize its sensitivity and specificity. This was used by the first author, with the help of a medical librarian, to search international electronic databases indexing medical (Cochrane Library, Medline, and EMBASE), Business (ABI/Inform, ASSIA, and Sociological abstracts), ICT (IEEE Xplore), and multidisciplinary (Scopus, Web of Science Core Collection, and ScienceDirect) research, as this topic lies at the intersection of informatics, management, and health. There were no restrictions on language or publication year applied to the literature search.

In addition to the electronic databases, various gray literature sources were also reviewed to identify relevant studies, using a subset of the search terms described in the next section. They included, but were not limited to 4 types of sources. First, WHO reports and working papers were searched using WHO’s *Institutional Repository for Information Sharing* and the following query: HRIS OR eHRM OR e-HRM OR “Human resource information system”. Second, with respect to professional, marketing, and consulting company reports, the resources/publications/web pages of the following organizations were examined individually to identify relevant articles: Chartered Institute of Personnel and Development, Society for Human Resource Management, Deloitte, Ernst & Young, PricewaterhouseCoopers, KPMG, Towers Watson, McKinsey & Company, Boston Consulting Group, and Sierra-Cedar. Third, white papers and publications of the Healthcare Information and Management Systems Society available at their Resource Library (under “Health IT” topics) were included. Finally, academic theses were identified via Google’s search engine using the following query: HRIS OR eHRM OR e-HRM OR "Human resource information system" AND Health AND Thesis. The first 7 pages of the returned results were considered relevant and reviewed. References of qualified articles were “snowballed” to identify other relevant studies. Once data extraction is complete, experts in the field will be consulted to identify any further relevant articles that may have been missed.

### Keywords Identification

A comprehensive list of keywords was created for this systematic review [39] through three steps. First, the following types of HR-related terms were identified: (1) HR terminology that the authors were familiar with based on their background readings; (2) terms that were returned when searching under “Human resource” in the US National Library of Medicine’s Medical Subject Headings (MESH) browser, such as staff and manpower; and (3) terms articulated within a highly cited expert review analyzing theoretical, methodological, and topical aspects of e-HRM, and in the abstracts of the referenced articles [21] (added during the “Search Query 2” development stage). Second, the ICT-related terms “information systems” and “information technology” were selected based on background readings. Third, general health terminology that the authors were already familiar with were identified: health, health care, hospital, clinic*, and medic*. Health care and care were not sufficiently sensitive. Searching the MESH terms database did not reveal any additional relevant results.

### Search Query Development

Three main search queries (Table 1) for the identified keywords were tested (Tables 2-5) by the first author on June 25-26, 2015. Their results were then shared with the review team to jointly identify the search query most likely to ensure the highest level of inclusion of relevant studies.

**Table 1:** Search query development.

	Search Query 1	Search Query 2	Search Query 3
Search query testing	Table 2	Keywords testing: Table 3 ^a^ Search query testing: Table 4	Table 5
Conclusions	We decided not to proceed with this option as it generated results that were too generic.	Additional keywords [21] were added to the search query. HR terms were combined with ICT terms and inverted commas were used for these combined keywords, so the search would show only relevant results. Insufficiently sensitive keywords that yielded 0 search results in all databases were not included in further searches (Table 3). Insufficiently specific keywords yielding large numbers of irrelevant results were also rejected (Table 3). Keywords yielding more than 5000 results from all databases per keyword are subclassified as “too broad” in Table 3. Total number of keywords included in the search query = 50 The search query was adopted individually for each electronic database. The total number of returns was 18,000, which included studies from various industries. Therefore, the review team jointly decided to limit the search to HRIS studies in health (Search Query 3).	The search query was adopted individually for each electronic database. Total number of returns = 6663

^a^Full keyword testing statistics for each database can be requested by email from the first author.

**Table 2:** Search query 1 testing.

Search query	Web of Science core collection	Scopus
((HR OR “HR management” OR “HR administration” OR “Human resource” OR “Human resource management” OR “Human resource administration*” OR Workforce OR “Workforce management” OR “Workforce administration” OR Personnel OR “Personnel management” OR “Personnel administration” OR Manpower OR “Manpower management” OR “Manpower administration” OR Employee OR “Employee management” OR “Employee administration” OR Staff OR “Staff management” OR “Staff administration”) AND (“Information technolog*” OR “Information system*”)) OR (“e-HRM” OR HRIS OR “Electronic Human Resource”)	7381	108,256

**Table 3:** Search query 2 keywords testing.

Status	Keywords
Included	“E-HR” OR “e-HRM” OR eHRM OR HRIS OR “electronic Human resource” OR “HR management system*” OR “HR information system*” OR “HR technolog*” OR “HR management information system*” OR “HR administration system*” OR “HR information technolog*” OR “HR management technolog*” OR “Workforce management system*” OR “Workforce information system*” OR “Workforce technolog*” OR “Manpower management system*” OR “Manpower information system*” OR “Manpower management information system*” OR “Employee management system*” OR “Employee information system*” OR “Employee management information system*” OR “Staff management system*” OR “Staff information system*” OR “Staff management information system*” OR “Staff administration system*” OR “Human resource information technolog*” OR “Human resource management technolog*” OR “Human resource* technolog*” OR “Human resource information system*” OR “Human resource management information system*” OR “Human resource administration system*” OR “Human resource management system*” OR “Personnel information system*” OR “Personnel management information system*” OR “Personnel administration system*” OR “Personnel management system*” OR “Personnel Staffing and Scheduling Information Systems” OR “electronic HRM” OR “Virtual HRM” OR “Web-based HRM” OR “HR Portal” OR “HR Online” OR “HR Intranet” OR “E-recruit*” OR “Electronic recruit*” OR “E-employment” OR “Virtual HR” OR “Web-based HR” OR “Business-to-employee” OR “Employee self service”
Not included (not sufficiently sensitive: no results)	“Human resource administration information system*”; “HR administration information system*”; “Workforce administration information system*”; “Personnel administration information system*”; “Manpower administration information system*”; “Employee administration information system*”; “Employee administration system*”; “Workforce administration system*”; “Personnel administration information system*”; “Manpower administration system*”; “Staff administration information system*”; “HR management information technolog*”; “Human resource administration information technolog*”; “Human resource administration technolog*”
Not included (not sufficiently specific: many irrelevant results)	“HR administration technolog*”; “Workforce management information system*”; “Human resource management information technolog*”; “Personnel technolog*”; “Personnel IT”
Not included (too broad: over 5000 hits for all databases per keyword)	“Personnel IS”; “Electronic learning”; “E-learning”; “Intranet”

### Article Screening and Selection

#### Procedure

Systematic review software (EPPI-Reviewer 4) is used to store, screen, and code all data generated by the search strategy citations. Two reviewers are screening and coding titles and abstracts for potentially eligible studies, for which full-text articles will be retrieved. In cases for which it is impossible to locate the full text of an article, requests will be sent directly to the authors. Reasons for exclusion will be noted for the remaining articles. A structured form will be used to summarize and classify the articles. Disagreements between reviewers will be resolved through consensus or arbitration by a third reviewer. A PRISMA flow diagram will illustrate the study selection process and ensure transparency of the review. Finally, content experts will be consulted to ensure that important articles have not been missed.

#### Inclusion Criteria

This study has two inclusion criteria. First, any study involving a formal or semiformal approach to the investigation or evaluation of HRIS, whether led by academia, industry (eg, consulting sector), or from within the health care sector will be included. This includes, but is not limited to, studies of HRIS development, implementation, deployment, diffusion, adoption, use, and impacts. Second, studies of broader nonclinical business/administrative/ERP systems that explicitly examine their application to HR practices will also be included.

#### Participants

There will be no exclusion based on participants/population group.

#### Interventions

This review is not restricted to intervention studies; however, it will include evaluations of interventions aimed at engaging personnel in the use of HRIS, as well as evaluation studies for which the implementation of HRIS represents an intervention, whether or not this is part of an explicit experimental design.

**Table 4:** Search query 2 testing.

Database	“E-HR” OR “e-HRM” OR eHRM OR HRIS OR “electronic Human resource” OR “HR management system*” OR “HR information system*” OR “HR technolog*” OR “HR management information system*” OR “HR administration system*” OR “HR information technolog*” OR “HR management technolog*” OR “Workforce management system*” OR “Workforce information system*” OR “Workforce technolog*” OR “Manpower management system*” OR “Manpower information system*” OR “Manpower management information system*” OR “Employee management system*” OR “Employee information system*” OR “Employee management information system*” OR “Staff management system*” OR “Staff information system*” OR “Staff management information system*” OR “Staff administration system*” OR “Human resource information technolog*” OR “Human resource management technolog*” OR “Human resource* technolog*” OR “Human resource information system*” OR “Human resource management information system*” OR “Human resource administration system*” OR “Human resource management system*” OR “Personnel information system*” OR “Personnel management information system*” OR “Personnel administration system*” OR “Personnel management system*” OR “Personnel Staffing and Scheduling Information Systems” OR “electronic HRM” OR “Virtual HRM” OR “Web-based HRM” OR “HR Portal” OR “HR Online” OR “HR Intranet” OR “E-recruit*” OR “Electronic recruit*” OR “E-employment” OR “Virtual HR” OR “Web-based HR” OR “Business-to-employee” OR “Employee self service”
Web of Science core collection	952
Scopus	2482
ScienceDirect^a^	278
ABI/Inform^b^	6771
ASSIA	37
Sociological abstracts	58
Cochrane Library	16
Medline	542
EMBASE	227
IEEE Xplore^c^	1875+4762
Total	18,000

^a^Database was manually set to search in “Abstract, Title, Keywords” to match search strategies in other databases.

^b^Database was manually set to search “Anywhere except full text” to match search strategies in other databases.

^c^Database has limitations on the number of keywords; therefore, the search had to be run several times to ensure that all search query keywords were included.

#### Comparisons

This is not a review of clinical trials, and we anticipate that most studies will be of the qualitative/investigative type. However, for studies evaluating an intervention, relevant comparators will include baseline measures of efficiency. Examples include payroll processing time, and indicators of impact, such as staff absenteeism, which could theoretically be associated with workload, or patient morbidity, which may be theoretically associated with effective staff deployment. Our inclusion criteria encompass all types of research or evaluation.

#### Context

Any health organization, including primary, secondary, or tertiary care settings, or health systems where HRIS are implemented at scale may be included.

#### Exclusion Criteria

Reports that are purely descriptive, only concerned with the computational design aspects of systems, or pure market research will be excluded, as the aim of this project is to glean both softer and harder forms of evidence (eg, social studies, quality improvement projects, and impact evaluations).

### Outcomes

#### Primary Outcomes

Primary outcomes are measures of organizational efficiency, effectiveness, safety, quality, and cost effectiveness.

#### Secondary Outcomes

Secondary outcomes include the effectiveness of change management processes; indicators of implementation, adoption, and use; perceived benefits and disadvantages; perceived facilitators and barriers; and satisfaction of managers and/or employees.

**Table 5:** Search query 3 testing.

Database	(“E-HR” OR “e-HRM” OR eHRM OR HRIS OR “electronic Human resource” OR “HR management system*” OR “HR information system*” OR “HR technolog*” OR “HR management information system*” OR “HR administration system*” OR “HR information technolog*” OR “HR management technolog*” OR “Workforce management system*” OR “Workforce information system*” OR “Workforce technolog*” OR “Manpower management system*” OR “Manpower information system*” OR “Manpower management information system*” OR “Employee management system*” OR “Employee information system*” OR “Employee management information system*” OR “Staff management system*” OR “Staff information system*” OR “Staff management information system*” OR “Staff administration system*” OR “Human resource information technolog*” OR “Human resource management technolog*” OR “Human resource* technolog*” OR “Human resource information system*” OR “Human resource management information system*” OR “Human resource administration system*” OR “Human resource management system*” OR “Personnel information system*” OR “Personnel management information system*” OR “Personnel administration system*” OR “Personnel management system*” OR “Personnel Staffing and Scheduling Information Systems” OR “electronic HRM” OR “Virtual HRM” OR “Web-based HRM” OR “HR Portal” OR “HR Online” OR “HR Intranet” OR “E-recruit*” OR “Electronic recruit*” OR “E-employment” OR “Virtual HR” OR “Web-based HR” OR “Business-to-employee” OR “Employee self service”) AND (Health OR Healthcare OR Hospital* OR Clinic* OR Medic*)
Web of Science core collection	105
Scopus	838
ScienceDirect^a^	86
ABI/Inform^b^	731
ASSIA	20
Sociological abstracts	10
Cochrane Library	16
Medline	428
EMBASE	134
IEEE Xplore^c^	4295
Total	6663

^a^Database was manually set to search in “Abstract, Title, Keywords” to match search strategies in other databases.

^b^Database was manually set to search “Anywhere except full text” to match search strategies in other databases.

^c^Database has limitations on the number of keywords; therefore, the search had to be run several times to ensure that all search query keywords were included. Original number of returns was 4847. This result was then checked and cleaned of duplicates with the help of systematic review software (EPPI-Reviewer 4). In total, 552 duplicates were removed during this process.

### Data Abstraction

The following information will be extracted from each of the eligible studies: authors/institutional affiliation; year; setting (type of organization, country or region in which the study was conducted); innovation stage (eg, design, piloting, implementation); journal discipline; HRIS function/activity; research purpose/questions; theoretical basis (if specified, or if this can be deduced from the author’s description); study design; main findings; and conclusions. Other fields may be added as the analysis progresses.

### Data Analysis and Synthesis

It is expected that data synthesis will be descriptive, only due to the interdisciplinary nature of the review, variety of study designs, outcomes, and heterogeneity of technology functions and their applications. The data, therefore, will be descriptively summarized and narratively reported.

### Critical Appraisal Techniques

Two reviewers will independently assess included studies to eliminate the risk of selection bias; the advice of the third reviewer will be sought in cases of disagreement. We anticipate that most studies will be qualitative. In line with other systematic literature reviews of qualitative research [40,41], we will use the Critical Appraisal Skills Programme (CASP) qualitative checklist [42] to assess study quality.

## Results

The initial searches have now been completed and the results are being analyzed. The review is expected to be complete and published by the end of 2015.

## Discussion

This review aims to present an unbiased summary and analysis of the existing evidence on HRIS development, adoption, implementation, and impacts regarding health organizations worldwide. We believe that the review will provide a useful resource for decision makers and managers considering or already implementing HRIS. We also expect that the recommended directions for future research that will follow from this review will generate more discussion and research among scholars with interest not only in HRIS, but also in business and support systems in health care.

## Abbreviations

E-HR: electronic human resource
E-HRM: electronic human resource management
ERP: enterprise resource planning
HR: human resource
HRIS: human resource information system
HRM: human resource management
ICT: information and communication technology
MESH: Medical Subject Headings
WHO: World Health Organization
